# A step toward restoring hand functions in patients with multiple sclerosis—a study protocol

**DOI:** 10.3389/fresc.2023.1053577

**Published:** 2023-06-14

**Authors:** Maryam Zoghi, Shapour Jaberzadeh

**Affiliations:** ^1^ Discipline of Physiotherapy, Institute of Health and Wellbeing, Federation University, Melbourne, VIC, Australia; ^2^ Non-invasive Brain Stimulation & Neuroplasticity Laboratory, Department of Physiotherapy, School of Primary and Allied Health Care (SPAHC), Faculty of Medicine, Nursing and Health Sciences, Monash University, Melbourne, VIC, Australia

**Keywords:** multiple sclerosis, hand functions, rehabilitation, myelin sheath development, transcranial direct current simulation

## Abstract

Multiple sclerosis (MS) is a chronic autoimmune disease characterized by inflammation, demyelination of axons, and oligodendrocyte loss in the central nervous system. This leads to neurological dysfunction, including hand impairment, which is prevalent among patients with MS. However, hand impairment is the least targeted area for neurorehabilitation studies. Therefore, this study proposes a novel approach to improve hand functions compared to current strategies. Studies have shown that learning new skills in the motor cortex (M1) can trigger the production of oligodendrocytes and myelin, which is a critical mechanism for neuroplasticity. Transcranial direct current stimulation (tDCS) has been used to enhance motor learning and function in human subjects. However, tDCS induces non-specific effects, and concurrent behavioral training has been found to optimize its benefits. Recent research indicates that applying tDCS during motor learning can have priming effects on the long-term potentiation mechanism and prolong the effects of motor training in health and disease. Therefore, this study aims to assess whether applying repeated tDCS during the learning of a new motor skill in M1 can be more effective in improving hand functions in patients with MS than current neurorehabilitation strategies. If this approach proves successful in improving hand functions in patients with MS, it could be adopted as a new approach to restore hand functions. Additionally, if the application of tDCS demonstrates an accumulative effect in improving hand functions in patients with MS, it could provide an adjunct intervention during rehabilitation for these patients. This study will contribute to the growing body of literature on the use of tDCS in neurorehabilitation and could have a significant impact on the quality of life of patients with MS.

## 1. Introduction

Multiple sclerosis (MS) is a chronic autoimmune disease that primarily affects females between the ages of 20 and 40 years old (1). Pathologically, it produces lesions throughout the central nervous system (CNS) in both gray and white matter (2) with demyelination of axons and oligodendrocyte loss and resulting neurological dysfunctions.

MS affects over 33,335 people in Australia (3) and more than two million worldwide (4). The neurological symptoms in these patients depend on the exact neuroanatomical location of these plaques. In the first year of the disease, the most reported symptoms are impaired sensory function (85%), fatigue (81%), impaired hand function (60%), and mobility (50%) (5). Among these symptoms, impaired hand functions were the least targeted area for neurorehabilitation studies (6) despite the fact that in a survey of 360 patients with MS, 88% of respondents rated hand and arm functions as being more important to them than lower limb functions (7). Johansson et al. (2007), reported that 167/219 patients with MS [Expanded Disability Status Scale (EDSS) range = 0–9.5] had a disability in their manual dexterity (8). In 2016, MS trust foundation in UK formally launched an awareness campaign “*Think Hand*” to increase the attention of medical professionals and drug manufacturers to hand and arm functions in this group of patients. This proposal is designed to take a step toward developing a ground-breaking approach to “restoring hand functions in patients with MS”.

## 2. Motor learning, neuroplasticity and recovery post CNS injury

Neuroplasticity can be defined as the ability of the brain to change, adapt and reorganize in a new environment (9) which is one of the main mechanism for restoration of motor skills e.g., manual dexterity post CNS injury. Motor skill learning can be considered as the acquisition of new patterns of muscle activation in time and space to improve performance of a motor task (10).

Motor learning consists of several phases. An initial rapid improvement (phase 1) is followed by a consolidation period that lasts several hours (phase 2), and then a slow learning phase which proceeds with continued practice and leads to gradual improvements in performance (retention—phase 3) (11). Phase 1 involves unmasking of connections by disinhibition (11), and changes in the strength of synapses driven by afferent feedback (11). Mechanisms behind the consolidation of synaptic plasticity (phase 2) include long-term potentiation (LTP) and long-term depression (LTD). The slow later stage (phase 3) may involve synaptogenesis and new functional connections (10). This learning-dependent plasticity plays an important role in functional recovery from CNS injury. LTP, LTD and formation of new synapses are all seen in M1, which is considered a crucial site for motor learning (10).

Myelin is the sheath that surrounds and insulates axons throughout the CNS, increases the speed of electrical communication among neurons and is synthesized by oligodendrocytes. It has been shown that in the adult brain, production of oligodendrocytes and myelin is a continuous process (12) which is necessary for learning new motor skills and also important for the process of neuroplasticity (13). McKenzie et al. (2014) showed the synthesis of new oligodendrocytes when mice were learning a new complex task. When the production of these cells was blocked before learning the task, the mice were not able to learn the task anymore. However, when the production of these cells was blocked after the learning process was completed, the mice still could recall the pre-learned task and complete the task with no problem (13). The researchers concluded that the generation of new oligodendrocytes is important for learning new motor skills. The structural changes in white matter were also shown in humans after practicing new motor skills such as playing piano (14) and juggling balls (15). MRI studies showed that activity-dependent myelo-modulation might underlie behavioural improvements by altering conduction velocity and synchronisation of nervous signals (16).

It has been argued that practicing a new motor skill triggers the formation of new neuronal connections or the strengthening of the existing connections in response to “repeating a particular sequence of movement”. The increased electrical activity in these circuits with practice will trigger the production of new oligodendrocytes and new myelination will make these connections stronger (17).

## 3. Augmenting the use-dependent plasticity in M1 and motor learning consolidation by transcranial direct current stimulation

Transcranial direct current stimulation (t-DCS) is a non-invasive technique that involves the application of weak, direct currents (1–2 mA) to the scalp via sponge-based rectangular pads (nominally 25–35 cm^2^) (18). This produces a sub-sensory level of electrical stimulation, which is imperceptible to most people during its application. In a small percentage of participants, it may cause minimal discomfort with a mild itching sensation, which usually disappears after a few minutes (19). The nature of these modulations depends on tDCS polarity. Using certain stimulation parameters, application of positive electrode (anode) over M1(a-tDCS) can increase; while application of negative electrode (cathode) over M1 (c-tDCS) can decrease the cortical excitability (20).

The immediate effect of tDCS occurs via altering ion concentrations in the extracellular space (21). However, pharmacological studies have revealed that the longer-lasting effects of tDCS are dependent on changes in neurotransmitter receptor function. There is an increasing body of evidence from pharmacological studies suggesting possible mechanisms contributing to the associated polarity-specific modulation of cortical plasticity. There is evidence for both GABAergic and dopaminergic modulation of tDCS-induced effects (22). Both the anodal facilitation and cathodal inhibition in M1 area are blocked by the N-methyl-d-aspartate (NMDA)-receptor antagonist dextromethorphan (20). Stagg et al. (2009) showed that facilitatory anodal stimulation leads to a significant decrease in the GABA concentration in the cortex. In contrast, inhibitory cathodal stimulation leads to a significant decrease in glutamate (23). It has been shown that co-application of neuropharmacologically active drugs may prolong or even reverse stimulation effects (20, 24).

TDCS-induced effects can be extended after the duration of stimulation by changing the efficacy of NMDA receptors (25). The long-term induced effects of tDCS may include formation of new synapses, which is necessary for the induction and maintenance of neuroplastic after-effect excitability enhancement by tDCS (25). These modulatory effects on cortical excitability, neurotransmitters and LTP mechanism are key elements for learning and memory processing which can be used for therapeutic purposes (26).

Over the past two decades, tDCS has been used as a neuromodulatory technique to enhance motor learning and motor functions in health and disease in human subjects (27). A-tDCS can be used as a stand-alone intervention (28) or as an add-on technique to prime the effects of other training methods (29). As tDCS induces a non-specific effect, it is generally agreed that a-tDCS-dependent behavioural gains can be optimized with concurrent behavioural training. Literature indicates that the application of tDCS during motor learning can have priming effects on the LTP-like mechanism of its action and prolong the effects of motor training in health and disease (30). This concurrent application of a-tDCS and motor training may also strengthen glutamate receptor learning-dependent activity, selectively boosting training-dependent activation of specific neural networks and promoting motor learning consolidation (31).

O'Brien et al. (2018) completed a systematic review and meta-analysis on the effect of a-tDCS on fine motor skills in patients after stroke (32). They examined the data of 351 stroke patients and showed moderate improvement of manual dexterity in this population when tDCS was applied alone and large improvement when it was concurrently applied with another intervention (32).

TDCS is a safe intervention with no or minimal side effects (19). However, the induced excitability changes and the length of lasting effect of tDCS strongly depend on the electrode montage and parameters of stimulation: *intensity* and *duration* of stimulation (33). The effects of a-tDCS can be prolonged by using higher intensities or by increasing the duration of its application. High-intensity stimulation can affect different neuronal populations compared with low intensity stimulation. By increasing the intensity, the current may reach deeper sites that might not be the intended target. It has been shown that longer application of a-tDCS may cause excitability diminution (34). This might be caused by a calcium overﬂow-caused neuronal counter-regulation (34). Therefore to increase the length of lasting effects, other characteristics of a-tDCS application should be considered. Within-session repetition of a-tDCS is another alternative for prolongation of the a-tDCS lasting effects. The efficacy of this technique has been already tested on healthy participants (35).

We are aiming (for the first time) to train patients to learn a completely new motor skill with their fingers rather than practicing the pre-injury well-learned tasks repeatedly to trigger the production of oligodendrocytes and myelin in the motor areas of their brain. We are also aiming to facilitate this learning process by combining the practice sessions with concurrent application of a-tDCS.

### 3.1. Aim

To assess whether applying within session repeated anodal transcranial direct current stimulation (a-tDCS) on primary motor cortex (M1) during learning of a new motor skill can improve hand functions in patients with MS more than conventional neuro-rehabilitation strategies for hand function improvement.

**Hypothesis 1:** Learning a new motor skill will be more effective in improving hand functions compared to the current hand training in patients with MS.

**Hypothesis 2:** In patients with MS, applying within-session repeated a-tDCS on M1 while learning a new motor skill for five days can improve hand functions more effectively than learning the new motor skill alone or with sham stimulation.

**Hypothesis 3:** As the protocol for within session repeated a-tDCS has accumulative and long-lasting effects on cortical excitability in M1 area, continuing hand training sessions during weeks 2–8 in the experimental group will be more effective in improving hand functions compared to patients in sham group or learning a new motor skill alone.

## 4. Research plan

### 4.1. Design

The is a randomized controlled trial.

### 4.2. Participants

Seventy two patients with secondary progressive MS will be recruited. These patients will be assessed by a rehabilitation physician against the inclusion and exclusion criteria. Eligible participants will then be enrolled in the study.

**Inclusion criteria: (**a) 18 years of age and over; (b) secondary progressive MS; (c) impaired dominant hand functions (right side); (d) EDSS = 3–6 and (e) able to understand, speak and write in English.

**Exclusion criteria:** (a) skin conditions (e.g., eczema, lesions) on scalp; (b) metal inside the head (outside the mouth) such as shrapnel, surgical clips, or fragments from welding or metalwork; (c) any implanted devices such as cardiac pacemaker, cochlear implant, medical pump, or intracardiac line; (d) frequent or severe headaches; (e) previous head injury and any other brain related disease and (f) pregnancy and breast feeding.

### 4.3. Experimental design

**Figure 1:**
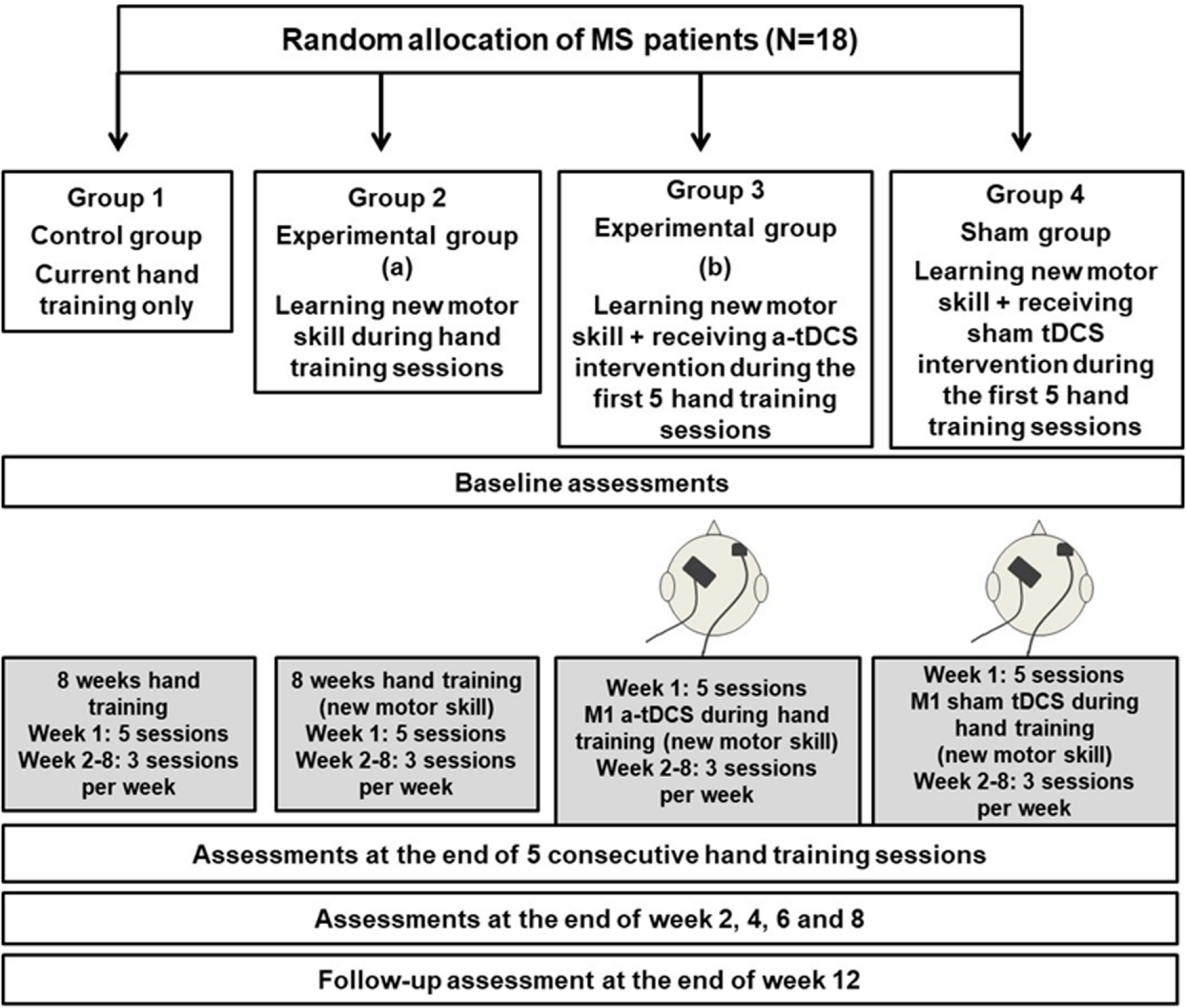
Flow diagram of randomization, intervention and assessments. This figure shows the randomization of the participants into 4 groups and the allocated intervention and assessment sessions throughout the trial. MS: Multiple Sclerosis; tDCS: Transcranial direct current stimulation.

Enrolled patients will be randomly allocated to one of four groups (*n* = 18 per group). Randomisation will be controlled by one of the investigators using a secure web-based computer-generated sequence which is a commonly used and reliable method for ensuring that study participants are assigned to different groups in a fair and unbiased manner (Figure 1).

**Group 1: Control group** (*n* = 18): Patients in this group will participate in hand training sessions only based on the current standard in clinics for 8 weeks. During week 1 this will be provided in 5 sessions over 5 consecutive days (30 min with rest) and during weeks 2–8, participants will have 2 sessions per week. They will have the following assessments: baseline assessments (before training); assessments during hand training (week 1, at the end of 5 consecutive training sessions and end of week 2, 4, 6 and 8); and follow-up assessments (week 12).

**Figure 2:**
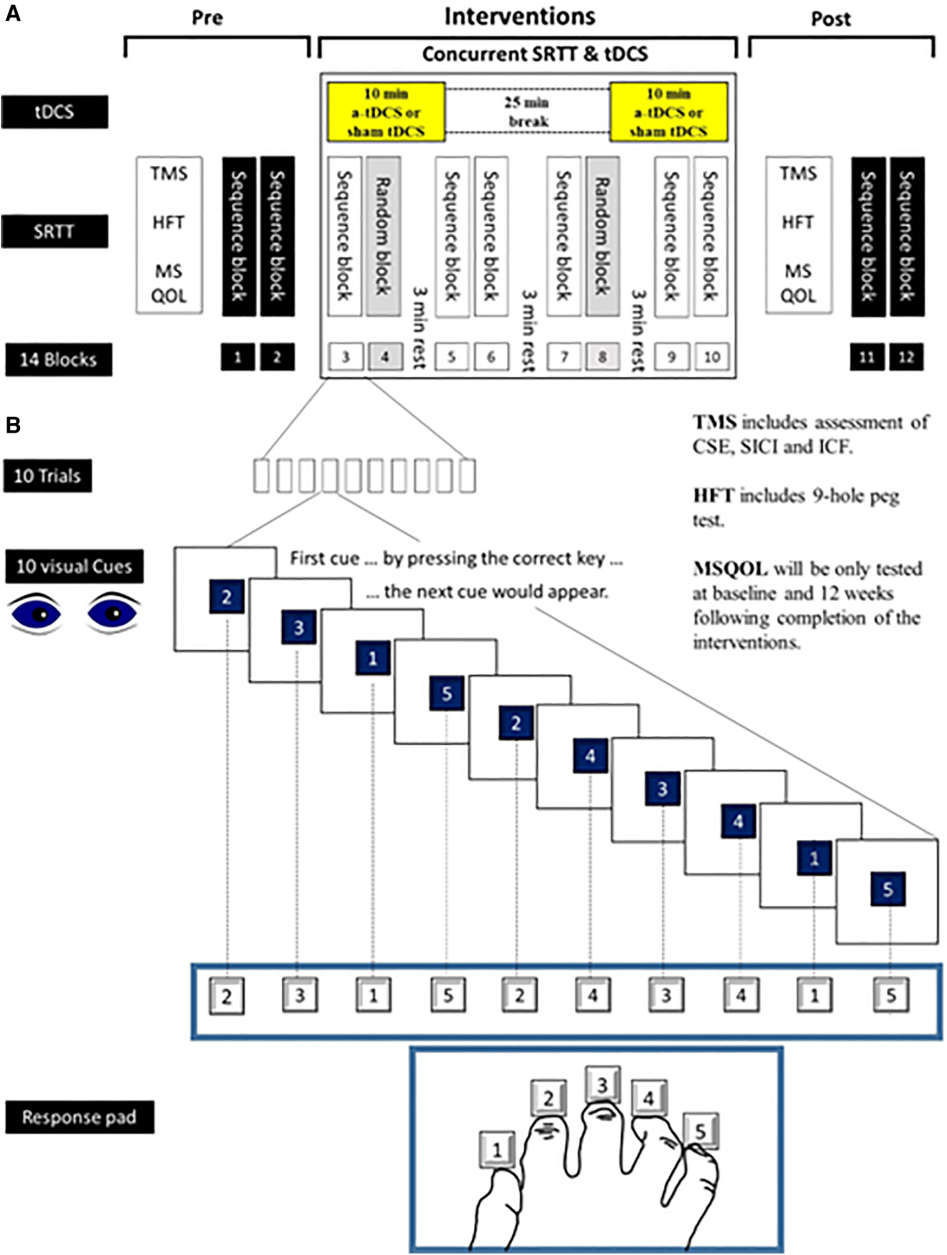
New motor skill—serial reaction time task (SRTT). This figure shows how SRTT will be used as a new motor skill during hand training sessions in this study. Numbers from 1 to 5 will appear on a computer screen and the subjects will be instructed to press the key with the finger that corresponds to that number as fast as possible. After a button is pushed, the number will disappear. The next number will be displayed 500 ms later. The learning test consists of 8 blocks (10 trials in each block). Each trial consists of 10 numbers which will appear with two types of sequence. In blocks 4 and 8 the sequence of numbers will be in a pseudo-random order in a way that each number will be presented equally frequently and never in the same order in two subsequent trials. In blocks 3, 5−7 as well as in blocks 9 and 10, the same sequence of numbers will be repeated 10 times (10 trials) (e.g., 2315243415). Subjects will not be told about the repeating sequence. SRTT, Serial Reaction Time Task; tDCS, Transcranial Direct Current Stimulation; TMS, Transcranial Magnetic Stimulation; CSE, Corticospinal excitability; SICI, Short Interval Cortical Inhibition; LICI, Long Interval Cortical Inhibition; ICF, Intracortical Facilitation; HFT, Hand Function Test; MSQOL, MS Quality of Life.

**Group 2: Experimental group (Hand training only**) (*n* = 18): Patients in this group will participate in hand training sessions in the exact time frame as Group 1 but they will practice a new motor skill (Figure 2) during their hand training sessions. The time frame for the assessments will be exactly the same as for Group 1.

**Group 3: Experimental group (a-tDCS)** (*n* = 18): Patients in this group will participate in hand training sessions similar to Group 2 while they will receive a-tDCS on M1 area concurrently during the first 5 sessions of their hand training in week 1. The time frame for the assessments will be exactly the same as the previous groups.

**Group 4**: **Sham group** (*n* = 18): Patients in this group will participate in hand training sessions similar to Groups 2 and 3 while they will receive sham tDCS on M1 area concurrently during the first 5 sessions of their hand training in week 1. The time frame for the assessments will be exactly the same as the other groups.

### 4.4. Rationale for the sample size

The study is a within-subject, repeated measures study with one between factor (Group) with four levels and one within factor (Time) with seven time points. As we are predicting an effect size of 0.30 (medium to high), 16 subjects per group (a total of 64 subjects) is needed to achieve 80% power with a 5% significance level. Allowing for a 10% drop out rate, each group will need 18 subjects.

### 4.5. A-tDCS intervention

A DC-Stimulator (neuroConn. DC Stimulator plus) will be used to deliver 1 mA current to the M1 area via two surface electrodes with pockets of saline soaked sponges (0.9% NaCl). Active electrode (anode) measuring 3 × 4 cm will be placed over the left C3 or C4 according to the International 10–20 EEG system for electrode placement. The return (cathode) electrode (5 × 7) will be placed over contralateral supraorbital area. Participants will receive within-session repeated a-tDCS or sham tDCS paradigm (10-25-10 protocol) for 5 consecutive days. 10-25-10 protocol involves 10 min of tDCS intervention, followed by 25 min of rest, and then a further 10 min tDCS. The stimulator software has a study mode for blinding purposes that encodes sham and active stimulation parameters and can be set individually using the software. The safety and feasibility of this protocol and also its long-lasting effect in these patients have been assessed in our previous study (36).

#### 4.5.1. Hand training session for group 1

The patients in this group will be assessed by a physiotherapist and an individualized hand training program will be written based on the current practice standard.

#### 4.5.2. Hand training session for groups 2–4

The participants will be seated in front of a computer screen at eye level behind a response pad with four buttons (numbered 1–4) and will be instructed to push each button with a different finger of the right hand (Thumb for button 1, index finger for button 2, middle finger for button 3, ring finger for button 4 and little finger for button 5) (Figure 2).

Numbers from 1 to 5 will be appeared on a computer screen and the subjects will be instructed to press the key with the finger that corresponds to that number as fast as possible. After a button is pushed, the number will disappear. The next number will be displayed 500 ms later. The learning test consists of 8 blocks (10 trials in each block). Each trial consists of 10 numbers which will appear with two types of sequence. In blocks 4 and 8 the sequence of numbers will be in a pseudo-random order in a way that each number will be presented equally frequently and never in the same order in two subsequent trials. In blocks 3, 5–7 as well as in blocks 9 and 10, the same sequence of numbers will be repeated 10 times (10 trials) (e.g., 2315243415). Subjects will not be told about the repeating sequence.

**Figure 3:**
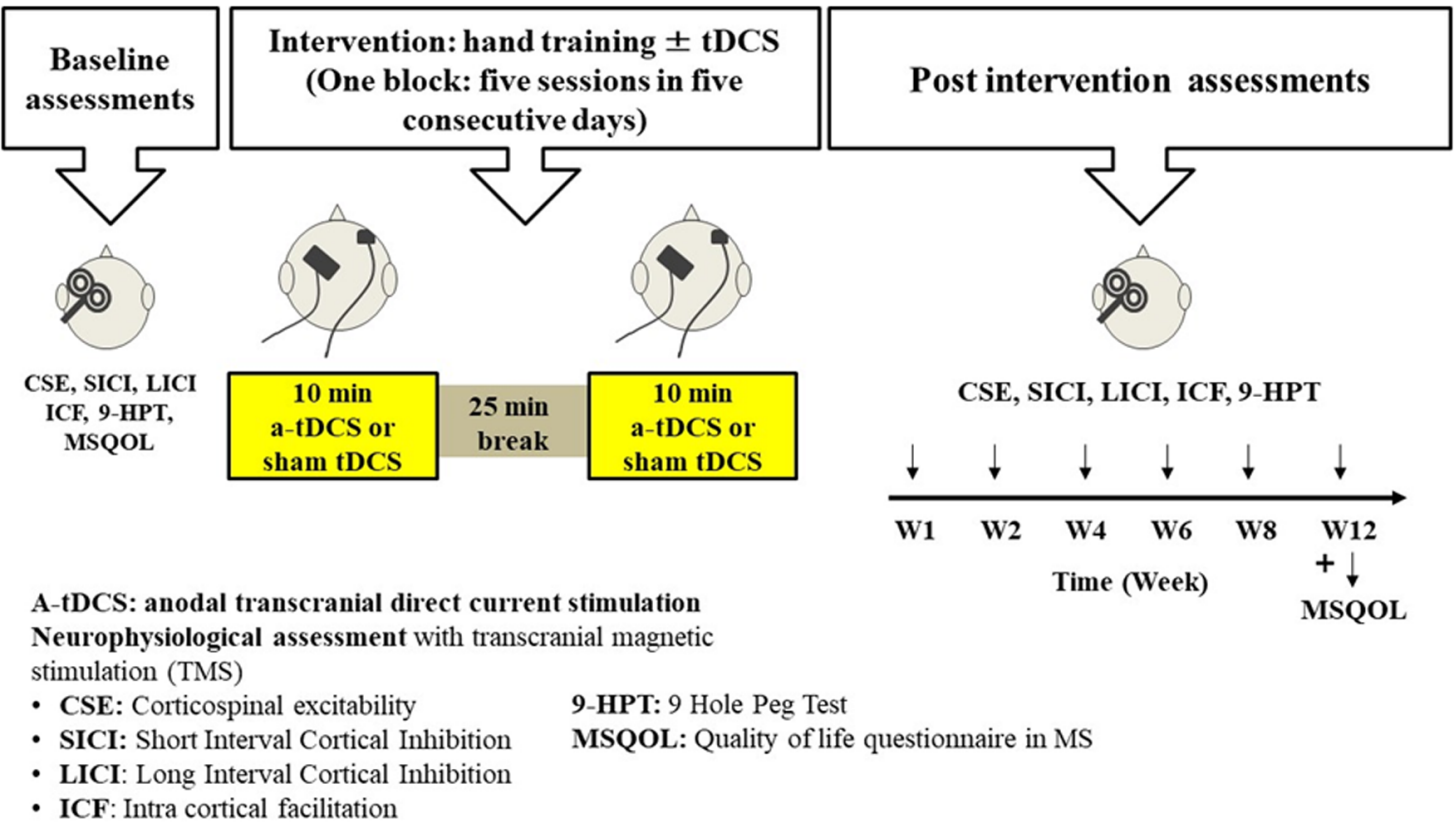
Timeline for intervention and assessments for one participant. This figure shows the timeline for the allocated intervention and assessment sessions for a participant in this study. Baseline assessment will be completed for each participant and then they start the hand training sessions ± tDCS intervention (5 sessions during week 1). During weeks 2−8, participants will have 2 hand training sessions per week. Then at the end of weeks 1, 2, 4, 6, 8 and 12, they will go through some post-intervention assessments or follow-up assessments.

This study will enable the investigation of the effect of learning a new motor skill in M1 in patients with MS on improvement of hand functions compare to the current hand training programs. It will also enable the investigation of the lasting effects of multiple applications of a-tDCS on cortical excitability of M1 area in patients with MS which can facilitate the motor skill learning process and in turn increase the functional outcome of the hand training sessions (Figure 3).

#### 4.5.3. Blinding

Participants in Groups 3 (experimental a-tDCS) and 4 (sham tDCS) will be blinded as to the tDCS intervention they will receive. The treatment/sham mode will be set through the software based on the group allocations.

## 5. Assessments: primary outcome measure

### 5.1. Manual dexterity: 9 hole peg test (9-HPT)

The 9-HPT is considered as a gold standard measure of manual dexterity and most frequently used in MS research and clinical practice (37). During this test patients will sit behind a desk and will be instructed to pick up one peg at the time from the container and place it in a hole as fast as they can, then take them off and put them back to the container again. Their completion time will be recorded for both hands in two consecutive trials.

### 5.2. Action research arm test (ARAT)

The Action Research Arm Test (ARAT) is a comprehensive assessment tool for evaluating upper limb function. The test takes around 10 min to administer.

The test consists of 19 items grouped in 4 categories: grasp, grip, pinch, and gross arm movement. Each item is rated on a 4-point scale ranging from 0 (no movement possible) to 3 (movement performed normally). The maximum achievable score is 57 (38).

### 5.3. Impact on participation and autonomy

Impact on Participation and Autonomy questionnaire is used to measure how an individual perceives their level of autonomy and participation in various domains, including outdoor autonomy, indoor autonomy, family role, social relations, and paid work/education. It evaluates both the perceived level of participation and the presence of problems in each domain (39).

## 6. Secondary outcome measures

### 6.1. Neurophysiological changes

Changes in the cortical excitability, intracortical inhibitory (GABAergic) and excitatory (glutamergic) interneurons in M1 area will be assessed by single or paired-pulse transcranial magnetic stimulation (TMS) (Magstim Bistim^2^) in a block of 20 stimuli. The motor evoked potentials (MEPs) will be recorded from the right first dorsal interosseous (FDI) muscle. To measure the resting motor threshold (RMT), MEP threshold will be tested in steps of 2% maximum stimulator output, and defined as the lowest intensity for which three of five successive MEPs exceed 50 µV (rest) peak-to-peak amplitude (40).

To assess the overall cortical excitability of M1, recruitment curve will be produced by single pulse TMS and a range or suprathreshold intensity (0.6 × RMT–1.2 × RMT).

Paired-pulse TMS will be used to deliver two pulses with interstimulus interval of 3 ms, 10 ms or 150 ms through a figure of eight coil to assess the function of the intracortical inhibitory (GABAergic) and excitatory (glutamergic) interneurons respectively. This inhibition or facilitation are termed short interval intracortical inhibition (SICI), Long interval intracortical inhibition (LICI) or intracortical facilitation (ICF). The first stimulus for measuring SICI and ICF is subthreshold (0.8 x resting motor threshold: conditioning stimulus), however, it is suprathreshold [strong enough to produce 0.8–1.2 mV MEP (peak to peak)] for measuring LICI. The second pulse is suprathreshold for all conditions {[strong enough to produce 0.8–1.2 mV MEP (peak to peak): test stimulus]} for producing MEPs in the right FDI.

The first pulse that is subthreshold or suprathreshold for a motor response in right FDI, activates the inhibitory or facilitatory circuits and reduces or increases the size of the MEPs elicited by a supra-threshold test TMS pulse delivered 3 ms, 10 ms or 150 ms later to measure SICI, ICF and LICI respectively (41). The area of the conditioned and unconditioned MEPs will be measured from the averaged rectified MEPs obtained in each trial (42). The size of the conditioned MEPs will be expressed as a percentage of the unconditioned test MEPs in order to assess the effectiveness of SICI and ICF.

### 6.2. Multiple sclerosis quality of life-54 (MSQOL-54)

The MSQOL-54 is a multidimensional health-related quality of life measure. It consists of the Short Form 36 (SF-36) (43) along with 18 additional items pertinent to people with MS. The 36 items grouped into 8 domains: physical function, social function, physical role limitations, emotional role limitations, pain, energy/fatigue, mental health, and general health.

## 7. Statistical analysis

A two-way repeated measures ANOVA will be undertaken to analyse the data. For the primary analysis, the effects of the main factors “Group (4 groups)” and “Time (7 time points)” and the interaction of “Group * Time” will be assessed on HPT scores. Prior to analysis, tests for normality will be undertaken and, if the assumption of normality is violated, a Wilcoxon rank-sum test will be used. For the secondary analyses, similar analyses will be performed for outcomes (HPT scores, quality of life, CE, SICI, LICI and ICF) at pre intervention, post 5-days intervention, W2, W4, W6, W8 and W12.

## 8. Outcome

If learning and practicing a new motor skill shows more significant improvement in hand functions in patients with MS compared to practicing the pre-injury well learned upper limb and hand function tasks, this approach can be assessed through a clinical trial first and then be easily adopted by all clinicians as a new approach in restoring the hand functions in this population. Furthermore, if application of within-session repeated a-tDCS is shown to have cumulative significant effects in improving hand functions in people with MS, tDCS may provide a low-risk, non-invasive option as an adjunct interventions during rehabilitation for these patients.

## 9. Discussion

To reduce the impact of this disease on patients, their families, carers, and the aged care sector, we need to assist them in remaining independent for as long as possible (44). In this study we propose a number of initiatives for to have better outcomes for these patients.

We propose several initiatives in this study to improve treatment outcomes for these patients:

1. Choosing upper limb (hand functions) as the target of treatment
2. Considering a new training concept for treating manual dexterity
3. Using within session repeated application of tDCS
4. Using multiple session concurrent application of tDCS and training

The following sections will discuss each of these initiatives:

### 9.1. Upper limb as the target of treatment

Upper limb functions and specifically hand functions play an essential role in providing independence for these patients. It has been shown that manual dexterity is usually impaired in these population interfering with daily and social activities and are associated with loss of employment, decreased quality of life and increased health care costs (8, 45). It has been reported and suggested that “MS continues to represent a serious burden for people with MS and the community in terms of both economic impact and QoL. Interventions that slow or prevent the accumulation of disability in MS are likely to have a substantial impact on the economic costs and QoL of people with MS” (46).

In a systematic review, Lamers et al. (2016) reported that rehabilitation research targeting the upper limb functions in MS is rare compared to research targeting the lower limbs (6). A large number of different rehabilitation strategies have been applied to improve upper limb function in this population, ranging from resistance and endurance training on the body functions and structures level to task-oriented training on the activity level (6). It is impossible to determine the effectiveness of a specific rehabilitation strategy for upper limb function due to the diversity of strategies and included patients (EDSS: 1–8) in different studies.

### 9.2. Considering a new training concept for treating manual dexterity

**Figure 4:**
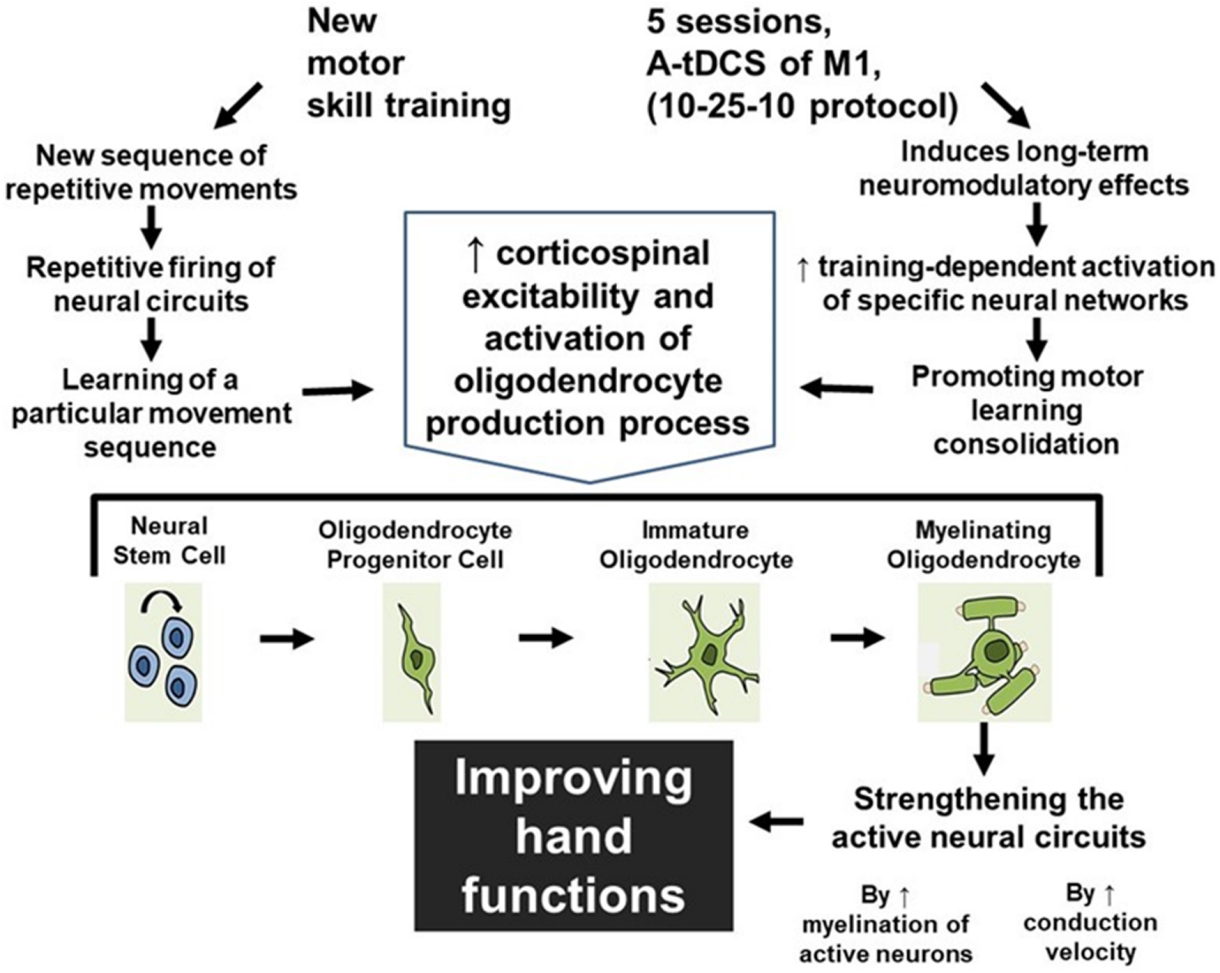
The accumulative effect of learning a new motor skill and receiving a-tDCS over M1 area. As a result of learning a new motor skill and receiving a-tDCS over M1 area, the possible mechanism for improving hand functions is shown in this figure.

We are proposing that patients should practice a completely new motor skill with their hand fingers rather than practicing pre-injury well-learned tasks repeatedly to trigger the production of oligodendrocytes in M1 area in their brain which will in turn trigger the production of myelin. This learning process and task-dependent neuroplasticity in M1 area will be facilitated by a new developed a-tDCS technique which provides accumulative long-term effect to maximize the effect of hand training sessions in restoring hand functions in patients with MS. (Figure 4).

### 9.3. Using within session repeated application of tDCS

It has been shown that, longer applications of a-tDCS are not the key for prolongation of the lasting effects on changes in corticospinal excitability. Indeed, a-tDCS lasting effects might be prolonged by repetition of shorter applications. A study by Bastani and Jaberzadeh (2014) indicated that within session repeated application of a-tDCS with a 25-minute interval significantly increased the magnitude of corticospinal excitability and motor performance. In contrast to a single 10-minute stimulation lasting for only 30 min or one hour, this increase lasted for 24 h.

### 9.4. Using multiple session concurrent application of tDCS and training

Greeley et al. (2020) investigated the effect of anodal tDCS on left M1 area during a discrete sequence task over three sessions (47). They showed that the anodal tDCS over M1 area facilitates motor sequence learning and faster re-learning after one year post intervention (47). Reis et al. (2009) in another study showed that application of anodal tDCS over M1 during learning of an isometric pinch force sequence task in 5 consecutive days produced greater effect on motor skill learning compared to offline learning and the effect could still be seen after 3 (48). They suggested that the observed long-term retention can be explained by plasticity-related protein synthesis in M1 area which can be promoted by using tDCS. This process was seen in non-human primates after successful learning a reaching task over several days (49).

If learning and practicing a new motor skill shows more significant improvement in hand functions in patients with MS compared to practicing the pre-injury well-learned upper limb and hand function tasks, this approach can be easily adopted by all clinicians as a new approach in restoring the hand functions in this population by triggering myelin synthetization. Furthermore, if application of within-session repeated a-tDCS is shown to have significant and cumulative effects in improving hand functions in people with MS, tDCS may provide a non-invasive option with little to no side effects as an adjunct intervention during rehabilitation period for these patients.

## Data Availability

The original contributions presented in the study are included in the article, further inquiries can be directed to the corresponding author.
